# Surface Engineering Design of Nano FeS@*Stenotrophomonas* sp. by Ultrasonic Chemical Method for Efficient U(VI) and Th(IV) Extraction

**DOI:** 10.3390/toxics11040297

**Published:** 2023-03-24

**Authors:** Zhongqiang Hu, Zhongkui Zhou, Jianping Guo, Yong Liu, Shunjing Yang, Yadan Guo, Liping Wang, Zhanxue Sun, Zhihui Yang

**Affiliations:** 1 State Key Laboratory of Nuclear Resources and Environment, East China University of Technology, Nanchang 330013, China; 2 School of Water Resources and Environmental Engineering, East China University of Technology, Nanchang 330013, China; 3 School of Environmental and Spatial Informatics, China University of Mining and Technology, Xuzhou 221116, China; 4 School of Metallurgy and Environment, Central South University, Changsha 410083, China

**Keywords:** microbial adsorption, microbial modification, nano-FeS, acoustic chemistry

## Abstract

Nano-FeS has great potential for use in the management of radioactive contaminants. In this paper, we prepared a FeS@*Stenotrophomonas* sp. composite material by ultrasonic chemistry, and it showed excellent removal of uranium and thorium from the solution. Through optimization of the experimental conditions, it was found that the maximum adsorption capacities for uranium and thorium reached 481.9 and 407.5 mg/g for a composite made with a synthetic ratio of 1:1, pH 5 and 3.5, respectively, for U and Th, and sonication for 20 min. Compared with those of FeS or *Stenotrophomonas* alone, the removal capacity was greatly improved. The results of a mechanistic study indicated that efficient removal of the uranium and thorium was due to ion exchange, reduction, and microbial surface adsorption. FeS@*Stenotrophomonas* sp. could be applied to U(VI) and Th(IV) extraction for radioactive water.

## 1. Introduction

The rapid development of the nuclear industry and the large-scale application of nuclear energy have led to the increased demand for rare earth resources [1,2]. Mining these resources has resulted in large amounts of radionuclides entering into environment in the form of wastewater, waste rock, and tailings [3,4]. Among them, the mobilities of uranium and thorium are closely related to their structural forms and changes in the pH. Complexation with organic matter make them acquire stronger in mobilization [5,6]. This greatly increases the risk of radioactive uranium entering the ecological food chain. The continuous accumulation of radionuclides in the human body causes a series of diseases such as leukemia and cancer [1,7,8]. Therefore, the prevention and control of radioactive contamination in water bodies have become major challenges common to mankind.

With the development of nanotechnology in recent years, the use of nanomaterials in wastewater treatments has gradually attracted attention. Due to their unique small size, the nanomaterials have large adsorption sites for binding metals, thus showing excellent adsorption capacity in practical applications [9,10]. Such as FeS nanoparticles, Li et al. [11] achieved a maximum adsorption capacity of 347.2 mg/g for U(VI) by using GO/FeS nanoparticles. From the perspective of environmental remediation, nanomaterials were highly valued for their low cost and high adsorption capacity [12]. Meanwhile, microbial methods demonstrate great potential for the treatment of water pollution. A previous study found that *Stenotrophomonas* sp. CICC 23833 exhibited high tolerance to radioactive elements as well as excellent removal capability. It also has a unique uranium detoxification mechanism [13]. Therefore, compared with traditional treatment methods, the microbial method has the advantages including strong self-propagation and environmental friendliness [14].

Combining nanomaterials with microorganisms for wastewater treatment significantly improves treatment efficiency [9]. Nanomaterials have more active sites due to their great specific surface areas, and microbial surfaces are rich in functional groups. Their combination of the above two increases the adsorption capacity of the composite material and overcomes the problems of agglomeration by the nanomaterials, low mechanical strengths of the microbial cells, and the difficult in solid–liquid separations of the materials [15,16]. Nanomaterials can also bring new properties to composite materials by their nature [17]. Ya Pang et al. [18] showed that polyvinyl alcohol, sodium alginate, and multi-walled carbon nanotubes, in combination with Pseudomonas aeruginosa, could reduce Cr(VI) levels with efficiencies of more than 90%. Huaqing Qin et al. [19] synthesized ZnS-cell composites by acoustic chemistry mothed for adsorption of Pb^2+^ and Cd^2+^. The results showed that, compared to the fungus, the removal efficiencies were increased by 140% and 160%, respectively. The above studies indicated that the combinations of nanomaterials and microorganisms enhanced the treatment of wastewater and were recyclable [20].

In this study, *Stenotrophomonas* sp. CICC 23833 was combined with FeS nanoparticles by using biological cells as templates with an acoustic chemical synthesis. This solved the problem of particle agglomeration by the nano-FeS materials and improved the adsorption capacity while improving the mechanical strengths of the microorganisms. Moreover, the nano-FeS increases the composite material better reduction capability, which promoted the reduction and fixation of U(VI) to U(IV) and further enhanced the treatment effect of radioactive elements in the water body.

This research aims to analyze the synthesis of FeS nanoparticles on bacterial surfaces. The adsorption performance of FeS-cells for radioactive elements was optimized with some parameters. Investigate the mechanism of adsorption of radioactive elements by FeS-cells. The combination of microorganisms and nanomaterials is expected to add a new dimension to the environmental treatment of wastewater contaminated with radioactive elements.

## 2. Materials and Methods

### 2.1. Materials

*Stenotrophomonas* sp. CICC 23833 was purchased from the China Industrial Microbial Strain Conservation Center (CICC). The bacteria were cultured for 36 h at 30 °C and 120 rpm in LB medium. Pure bacteria were obtained by centrifugation three times at 6000 rpm for 5 min [13]. U_3_O_8_ was purchased from the Beijing Institute of Metallurgy, Nuclear Industry, Beijing, China. The 1 g/L thorium solution was purchased from Beijing North Weiye Measurement Technology Research Institute Co., Beijing, China.

### 2.2. Synthesis of Nano-FeS on Microbial Surfaces

**Figure 1:**
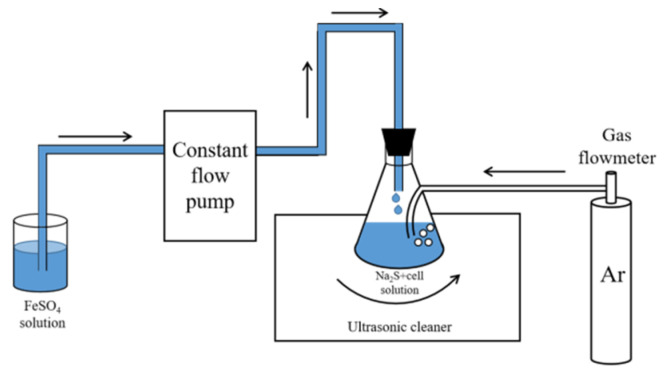
Schematic diagram of the preparation of FeS-cells complexes.

In this experiment, the nano-FeS layer was synthesized on the surface of *Stenotrophomonas* sp. by the sonochemical method [19,21]. Na_2_S·9H_2_O (0.120 g) was dissolved in 30 mL of deionized water and mixed completely with 20 mL of the pure bacterial solution made by the method described in Section 2.1. FeSO_4_·7H_2_O (0.139 g) was dissolved in 50 mL of deionized water and dripped into the mixture of Na_2_S and bacteria at a rate of 0.05 mL/s under argon while stirring well with a magnetic stirrer. The mixed solution was sonicated in an ultrasonic cleaner (600 W, 40 kHz) at room temperature. The treated mixed solution was centrifuged to obtain the FeS-cells complexes. Figure 1 shows a schematic diagram of the preparation of FeS-cells complexes.

### 2.3. Characterization

The synthesized FeS-cells complexes were analyzed by X-ray diffraction (XRD, BRUKER D8-A25, Billerica, Germany) to determine the physical phases of the samples. The morphologies of the cells and the nanospheres were observed by scanning electron microscopy (SEM, Zeiss sigma300, Oberkochen, Germany) and transmission electron microscopy (TEM, FEI Tecnai G2 F30, Hillsboro, OR, USA). Fourier infrared spectroscopy (FTIR, Thermo Nicolet iS 5 FT-IR, Waltham, MA, USA) was used to detect changes in the chemical species on the microbial surfaces. The oxidation states and binding energies were determined by XPS (XPS, Thermo Escalab 250Xi, Waltham, MA, USA).

### 2.4. Adsorption Experiments

The radioactive element adsorption experiments were carried out with dosages of 0.1 g/L, an initial uranium/thorium concentration of 50 mg/L, a temperature of 30 °C, a pH = 5/3.5, and stirring at 120 r/min. The effects of different initial uranium concentrations (10–150 mg/L) on the adsorption capacities of FeS-cells were investigated by adjusting different initial uranium concentrations. The optimal FeS-cells synthetic ratio was found by varying the synthetic ratio of nano-FeS to bacteria (0.067:1–1.3:1). The system was adjusted to different pH values (3–8) to determine the optimal pH for adsorption. The effect of temperature on adsorption was investigated by adjusting the incubation temperature (20–40 °C). The effect of dosage on the adsorption capacity was investigated by adjusting the different dosage levels (0.05–0.4 g/L). Different sonication times (0–180 min) were chosen to investigate the effects on the treatment capacity. Samples were taken at different times (0–24 h) to determine the effect of adsorption time on the treatment effect.

## 3. Results and Discussion

### 3.1. Characterization Materials

**Figure 2:**
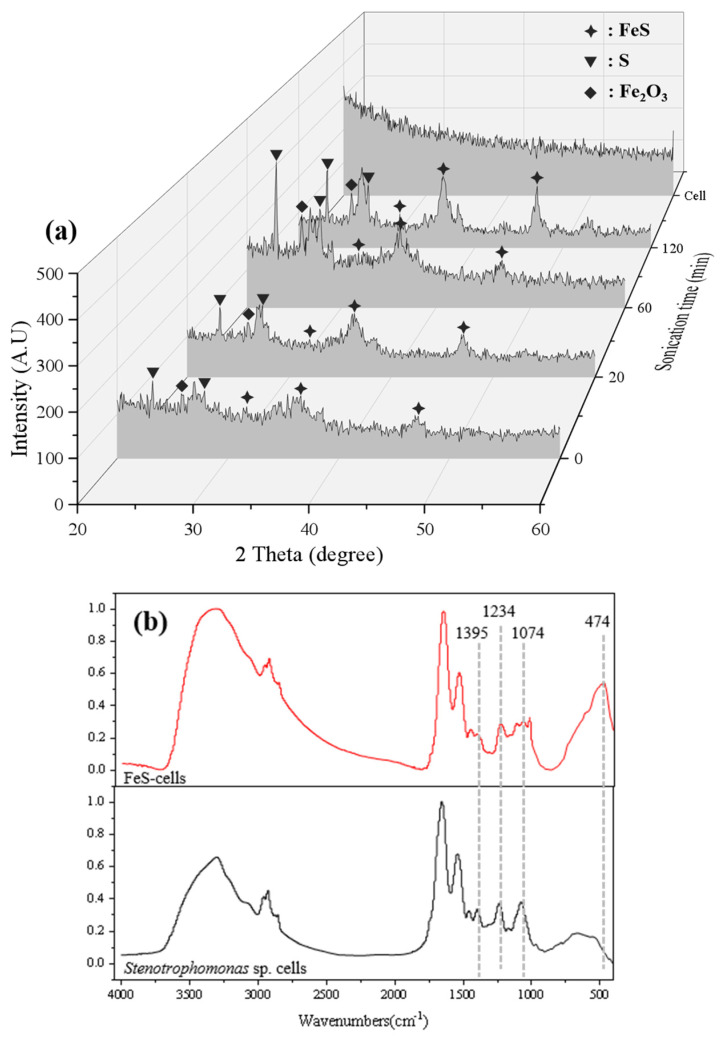
(**a**) XRD patterns of FeS-cells complex with different ultrasound reaction times. (**b**) FTIR maps of FeS-cells complexes and *Stenotrophomonas* sp. cell.

To detect the synthesis of nano-FeS, this experiment was performed by XRD analysis of FeS-cells complexes with different ultrasound times without considering the degree of cellular intactness. From Figure 2a, it can be seen that there is a large difference between the diffraction peaks of the FeS-cells complex and *Stenotrophomonas* sp. cells, which is caused by the large difference in the chemical composition of the two substances. The diffraction peaks of FeS were observed in the plots for the sonication times of 0, 20, 60, and 120 min, and the diffraction peaks were gradually obvious with the increase in the sonication time. These diffraction peaks correspond to the (100), (101), and (102) faces of the FeS standard XRD card (PDF#49-1632), where the lattice spacing of the (102) numbered crystal face is 0.19 nm. This proves the successful synthesis of FeS. Compounds such as S and Fe_2_O_3_ can be detected in the figure, which may be the product of small amounts of FeS oxidation. The FeS diffraction peaks appearing in the figure were weak, which might be caused by the small amount of FeS production or might be influenced by the interference of organic matter. Huaqing et al. also observed similar results in their study [19,22].

The infrared spectra of the cells before and after the sonochemical reaction were shown in Figure 2b. The partial peak increase after sonochemical synthesis was caused by the removal of impurities from the microbial surface by sonication and the same result was observed by Qin et al. [19]. The peaks at 1234 cm^−1^ and 1074 cm^−1^ correspond to the P=O and P−O vibrations of the carboxyl (−COOH) and phosphate groups [23,24]. The peaks at both sites diminished after sonochemical synthesis, suggesting that carboxyl and phosphate groups were involved in the synthesis of FeS on the bacterial surface. A new peak appears at 474 cm^−1^, which was reported by Duan et al., indicating that this peak was brought about by sulfide, which was consistent with the successful synthesis of FeS [10].

**Figure 3:**
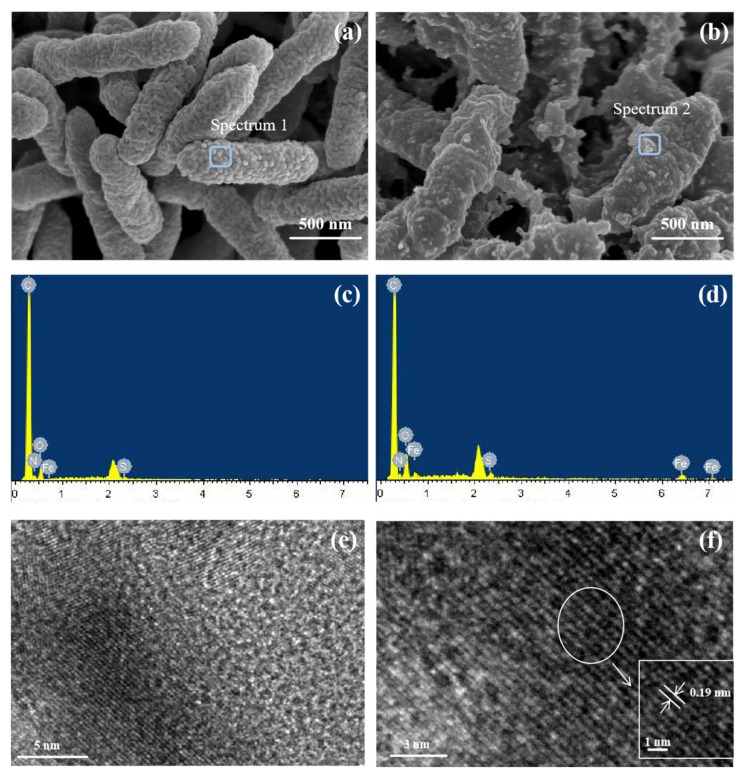
SEM and TEM images; (**a**,**c**) SEM-EDS images of *Stenotrophomonas* sp. (**b**,**d**) SEM-EDS images of FeS-cells (**e**,**f**) TEM maps of FeS-cells.

The cell morphologies of *Stenotrophomonas* sp. CICC 23833 before and after the sonochemical reaction are shown in Figure 3. The morphology of the bacteria was relatively intact, and the bacteria were not significantly damaged. The bacterial surface of the FeS-cells (Figure 3b) was rougher than that of the *Stenotrophomonas* sp. cell (Figure 3a), which may be due to the irregular distribution of the synthesized FeS nanoparticles on the cell surface [15]. This was evidenced by the EDS energy spectrum (Figure 3c,d), distinct Fe and S characteristic peaks appeared after the acoustic chemical reaction, all of which proved that FeS nanoparticles had been successfully synthesized on the surface of the bacteria.

The TEM morphology of the FeS-cells is presented in Figure 3e,f. The nano-FeS was irregularly distributed on the microbial surface in the form of nanoclusters with an average particle size of 40–50 nm, and the lattice distance of nano-FeS was 0.19 nm. Combined with previous XRD results, it further suggested that nano-FeS had been successfully loaded on the bacterial surface. Similar results were observed in the experiments of Dadong et al. [25].

### 3.2. Adsorption Properties

#### 3.2.1. Comparison of the Capacities of the Cells and FeS

**Figure 4:**
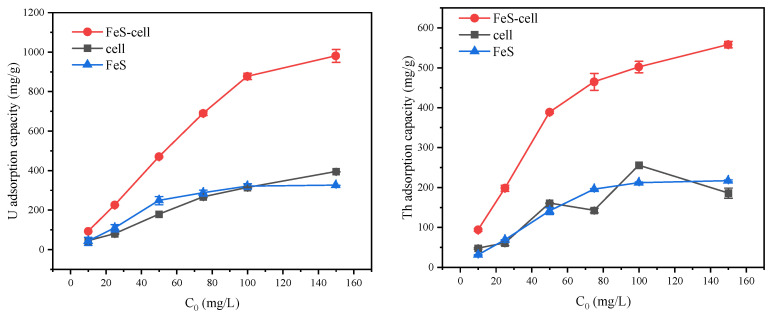
Comparison of the adsorption capacities of the FeS-cells, cells, and FeS at different initial concentrations.

We compared the U and Th adsorption energies of the *Stenotrophomonas* sp. cells, FeS, and the FeS-cells complexes. Figure 4 indicates that the FeS-cells had excellent adsorption capacities for both radioactive elements. At an initial concentration of 50 mg/L, the adsorption capacities for U and Th on FeS-cells were increased by 163% and 142%, respectively, compared to those of the *Stenotrophomonas* sp. cells. This was because the FeS brought more active sites to the cell surface, which led to the enhancement of the adsorption capacity. Moreover, FeS-cells were more widely on the microbial surface than FeS, which effectively avoided loss of the treatment capacity due to FeS agglomeration [15].

#### 3.2.2. Effects of the Synthetic Ratio of Nano-FeS and the Microorganisms

**Figure 5:**
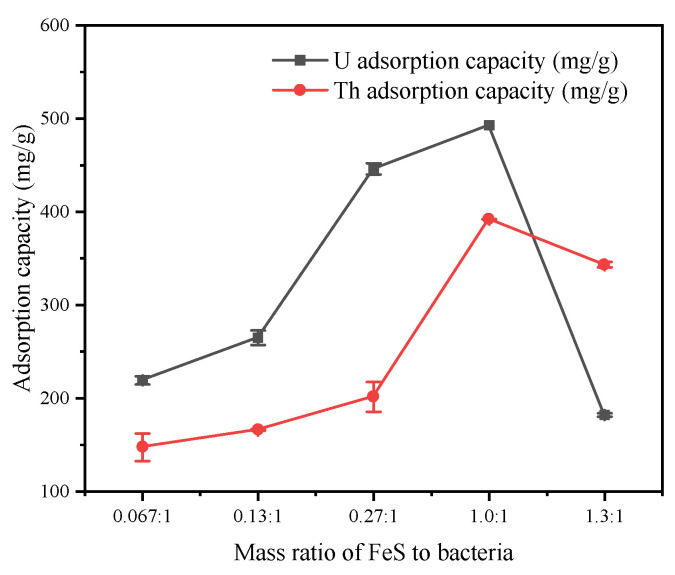
Adsorption capacities of the FeS-cells at different nan-FeS to bacteria synthetic ratios.

Since the removal abilities of the nano-FeS and bacterial cells were not excellent, the synthetic ratio of nano-FeS to bacteria was critical. The removal capacity was optimized at a mass ratio of 1:1 (Figure 5). This was attributed to the low mechanical strengths of the bacterial cells, the limited surface active functional groups, and therefore the poor removal capacities at lower ratios. The increased mechanical strength of the FeS-cells provided more active sites as the ratio was increased, thus increasing the removal capacity [10]. However, at higher ratios, the FeS nanoparticles tended to aggregate and cover the active sites, resulting in lower removal capacities [22,25].

#### 3.2.3. Effect of pH

**Figure 6:**
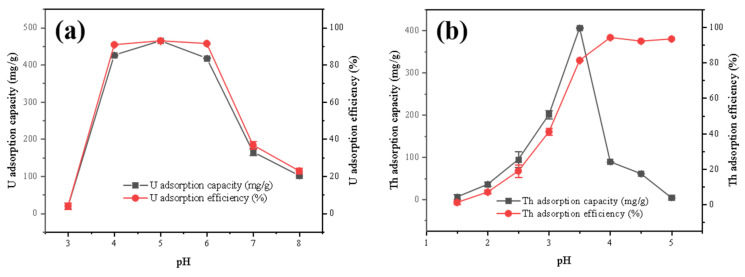
Effect of pH on adsorption of (**a**) U and (**b**) Th by the FeS-cells.

pH is a significant factor influencing the adsorption process. Figure 6 showed that the optimal pH for the removal of thorium was 3.5. Excellent removal of uranium was achieved within the pH range of 4–6. At low pHs, the H^+^ concentration was high, and the H^+^ competed with uranium and thorium for the limited active sites on the surfaces of the FeS-cells, leading to a reduced removal capacity [26]. Some research has indicated that FeS nanoparticles on the surface of bacteria were dissolved and reacted when the solution acidity was low, which also led to a reduction in the removal capacity [27,28]. At a high pH, uranium and thorium exist as hydroxides or precipitates, which could reduce the concentrations of the radioactive elements in the solution and reduce the removal capacities [27].

#### 3.2.4. Effect of Dosage

**Figure 7:**
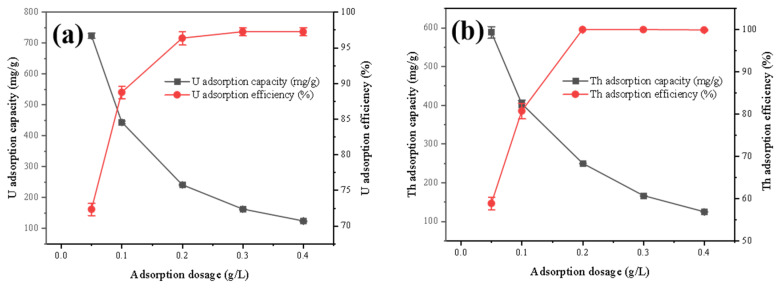
Effect of dosage on the adsorption of (**a**) U and (**b**) Th by the FeS-cells.

In Figure 7, the adsorption efficiencies of uranium and thorium are positively proportional to the FeS-cell dosing, while the adsorption capacity shows an opposite trend. This was attributed to the fact that as more FeS-cells were added, more active sites were available to remove uranium and thorium from the solution, and the adsorption efficiencies increased. In contrast, the pollutant loading per FeS-cell decreases with a higher dosage, which leads to a decrease in adsorption capacity.

#### 3.2.5. Effect of Ultrasonic Treatment Time

**Figure 8:**
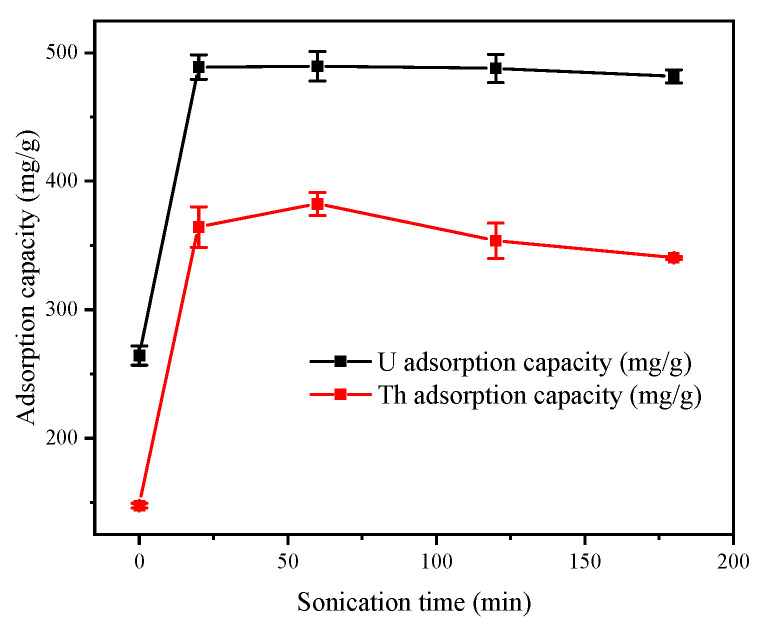
Effect of ultrasonication time on the adsorption capacity of the FeS-cells.

Ultrasound has gained considerable attention in the field of environmental remediation because of its high efficiency and other useful properties [29]. Figure 8 showed that the ultrasonically treated FeS-cells complexes exhibited a superior treatment. The adsorption capacities were not significantly changed by increasing the ultrasonic treatment times. This was because the ultrasound produced transient high-pressure and high-temperature local environments in the solution, which promoted the production rate and yield of the FeS nanoparticles, and the particle sizes of the FeS nanoparticles prepared with the assistance of acoustics were finer and more uniform [29,30]. Prior research has shown that ultrasonic removal of the passivation layer also led to large improvements in adsorption efficiencies [31]. This prevented the aggregation of FeS nanoparticles and produced more active sites for adsorption, which increased the adsorption capacity.

#### 3.2.6. Effect of Adsorption Time

**Figure 9:**
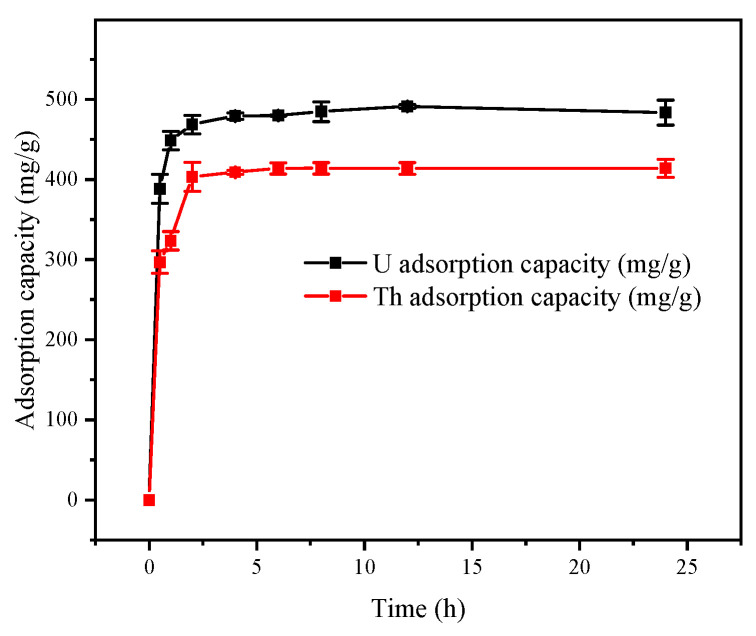
Effect of treatment time on the adsorption capacities of the FeS-cells.

The adsorption treatment time had a significant effect on the treatment effect, so the adsorption treatment time was also extremely important. Figure 9 showed that the adsorption by the FeS-cells was rapid in the first half hour when the uranium and thorium ions were bound to the active sites on the complex surfaces by electrostatic adsorption or complexation. This process also involved the ion exchange of the uranium and thorium elements [32,33]. After that, the adsorption rates slowed, which was caused by the complexation of the functional groups on the bacterial surface and the accompanying internal diffusion of the radioactive elements in the FeS-cells. After 2 h, adsorption equilibrium was reached, which was also the process of reducing radioactive elements with FeS-cells complex, and no significant desorption occurred within 24 h.

### 3.3. Adsorption Isotherms

The adsorption isotherm equation is used to describe the relationship between the solute concentrations in two phases when the solute reaches adsorption equilibrium at the interface of the two phases at a certain temperature. The Langmuir and Freundlich models are the most widely used empirical models for single solute adsorption systems [34]. The Langmuir model is typically used to describe single-molecule layer adsorption at homogeneous sites, and it indicates the relationship between adsorption and equilibrium concentration for a single-component system. The Freundlich model is more applicable to multilayer adsorption with nonhomogeneous surfaces [35].

**Figure 10:**
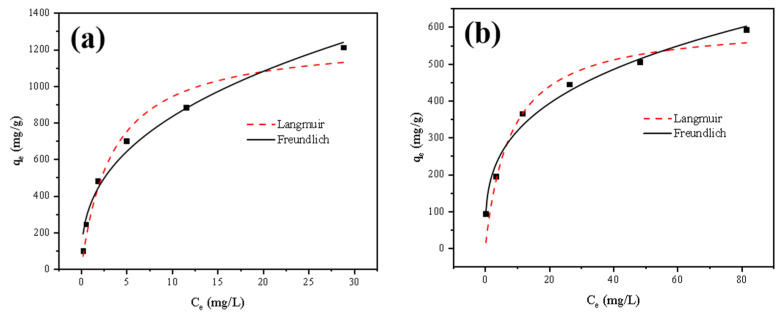
Isothermal model of adsorption of U^6+^ (**a**) and Th^4+^ (**b**) by the FeS-cells.

The previous experimental data were analyzed (Tables S1 and S2). As shown in Figure 10 and Table 1, the maximum adsorption amounts obtained in the Langmuir model (“U” and “Th” of 1263.7 and 610.9 mg/g, respectively) were similar to the experimental equilibrium adsorption amounts at the maximum set concentration. However, since the correlation coefficient of the Freundlich model was significantly higher, this theoretical adsorption amount did not represent the maximum adsorption amount of FeS-cells complexes. The values of 1/n for the Freundlich model were all below 0.5, and the 1/n values for thorium were lower than those for uranium. This indicated that the adsorption reaction was more likely to proceed spontaneously and that the adsorption of thorium was preferred [19,36]. The match between the Freundlich model and FeS-cells adsorption behavior also demonstrated the successful synthesis of nano-FeS on the bacterial surface, which caused a nonuniform distribution of adsorption sites [37].

### 3.4. Adsorption Kinetics

**Figure 11:**
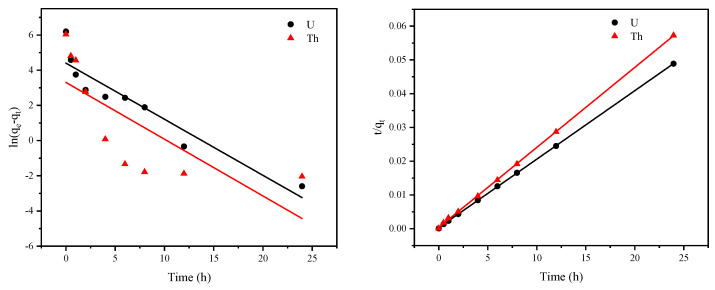
Pseudo-first-order and pseudo-second-order kinetic models for FeS-cells adsorption.

To investigate the transfer and kinetic mechanisms controlling the adsorption of radioactive elements by the FeS-cells complexes, the experimental data were fitted with pseudo-first-order, pseudo-second-order, and intraparticle diffusion models [15,38,39]. The fitting results for the pseudo-first-order and pseudo-second-order kinetic models are shown in Figure 11 and Table 2.

Based on the fitting coefficients, the fit of the pseudo-second-order kinetic model was better than that of the pseudo-first-order model in all cases, and the theoretical adsorption capacity (Q_e cal_) calculated with the pseudo-second-order model closely matched the experimentally derived adsorption capacity (Q_e exp_). This suggested that the chemisorption process better described the kinetics for U^6+^ and Th^4+^ adsorption by the FeS-cells complexes, which meant that the ability to adsorb uranium depended on the active site available on the bacterium and the concentration and nature of the uranium in solution [24].

**Figure 12:**
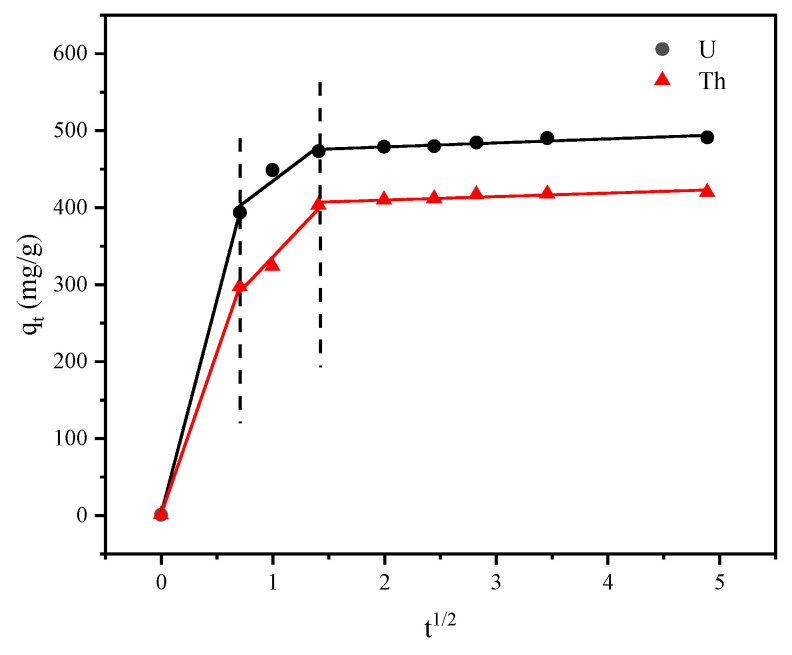
Kinetic model for intraparticle diffusion during FeS-cells adsorption.

We used the kinetic model for intraparticle diffusion to investigate the mechanism and adsorption rates. The results in Figure 12 with Table 3 exhibited multicollinearity. We divided the process into three steps. The first part involved contact of the U^6+^ and Th^4+^ with the nano-FeS and the functional groups on the surfaces of the bacteria, and ion exchange took place (U^6+^: Kd1 = 556.4 mg/(mg·h^1/2^), Th^4+^: Kd1 = 419.9 mg/(mg·h^1/2^)). The second part involved a smoother adsorption process, which corresponded to the diffusion of the U^6+^ and Th^4+^ within the pores of the FeS nanosphere on the bacterial surface or even to the cell interior for biosorption (U^6+^: Kd2 = 109.5 mg/(mg·h^1/2^), Th^4+^: Kd2 = 153.0 mg/(mg·h^1/2^)). In the third part, the adsorption process gradually approached equilibrium (U^6+^: Kd3 = 5.23 mg/(mg·h^1/2^), Th^4+^: Kd3 = 4.55 mg/(mg·h^1/2^)). From the kinetic parameters for intraparticle diffusion, the Ci increased gradually as adsorption proceeded, and C1 tended toward 0 in the first stage when the boundary layer effect was small and the intraparticle diffusion rate dominated [15,40]. As the concentrations of the radioactive elements in the solution gradually decreased, the K value also decreased [41].

### 3.5. Mechanism for U and Th Removal

**Figure 13:**
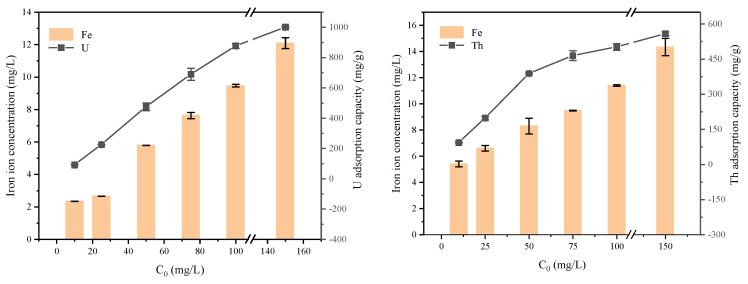
Adsorption capacities and ferric ion concentrations of the FeS-cells at different initial concentrations.

Numerous studies have shown the existence of ion exchange phenomena in the adsorption of FeS on radioactive elements [42,43]. To study the ion exchange behavior of FeS-cells complexes during adsorption, we examined the number of iron ions exchanged at different initial concentrations of U and Th. In Figure 13, as the adsorption amount increases and the concentration of iron ions in the solution increases, this indicates that the FeS nanosphere is involved in the removal of radioactive elements in the form of ion exchange [27,44]. The adsorption capacity of FeS-cells complexes was substantially increased compared to that of pure bacteria, indicating that FeS plays a larger contribution in the ion exchange during the removal of U and Th. In Figure S1, we found a linear correlation between the adsorption capacity of U and Th and the release of iron ions, but R^2^ did not strictly converge to 1. This is due to a combination of ion exchange and partial dissolution mechanisms, with similar results observed by Hyun et al. [44]. The molar mass difference between Fe and U and Th compared to each other is large, which results in the amount of iron ions undergoing ion exchange being much lower than the amount removed by U and Th [43].

**Figure 14:**
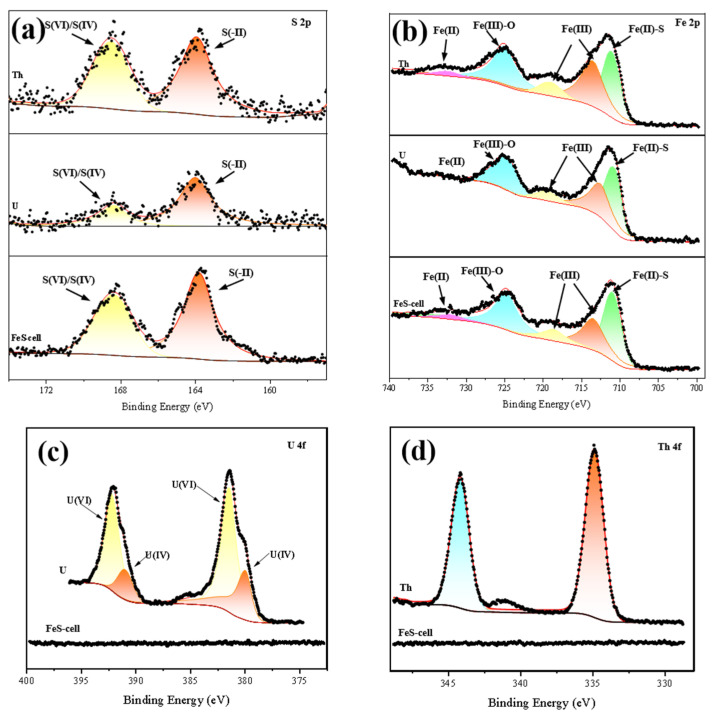
XPS patterns for the FeS-cells; (**a**) S, (**b**) Fe, (**c**) U, and (**d**) Th.

To understand the mechanism for interactions between the radioactive elements and the FeS-cells, the FeS-cells samples were characterized with XPS before and after adsorption. In Figure 14a, the S 2p spectrum contains two peaks near 163.8 eV and 168.4 eV, which corresponded to S(−II) and S(IV)/S(VI) in the FeS-cells complexes, respectively [40]. In Figure 14b, the Fe 2P spectrum has five peaks near 710.2, 713.48, 718.6, 724.79, 732.02 eV corresponding to ferrous sulfide, Fe(III), Fe(III)−O, and Fe(II), respectively [45,46]. In Figure 14c, the U 4f spectrum contained four peaks for U(IV) (380.0 ± 0.1 eV and 391.5 ± 0.1 eV) and U(VI) (382.4 ± 0.1 eV and 392.2 ± 0.1 eV) [25]. XRD results presented in Figure S2. These results showed the presence of two valence states for uranium adsorbed on the surfaces of the FeS-cells complexes and therefore the occurrence of uranium reduction during adsorption. The intensity of the peak corresponding to S(−II) in the S XPS spectrum was significantly reduced after uranium adsorption, which was caused by the participation of S^2−^ in the reduction in nano-FeS [10,47]. In addition, the intensity of the peaks corresponding to FeS and Fe(II) decreased and that of Fe(III) increased in the Fe elemental spectrum after uranium adsorption, indicating that some iron oxides may have been generated [40].

**Figure 15:**
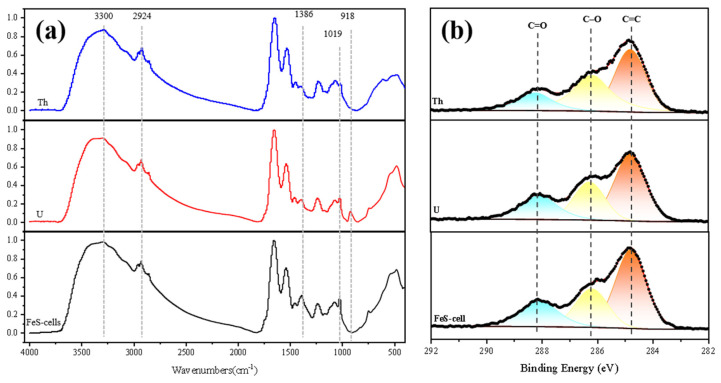
FeS-cells: (**a**) IR spectra; (**b**) C 1s XPS spectra obtained before and after U or Th adsorption.

The adsorption capacity of the bacterial surfaces in the FeS-cells complexes was indispensable. In Figure 15a, 3299 cm^−1^ corresposnds to the −OH stretching, where the diminished peak indicated the involvement of −OH in the adsorption process [10]. The peak weakening at 2924 and 2849 cm^−1^ was caused by the asymmetric stretching of the C−H bond of the −CH_2_ group with the C−H bond of the −CH_3_ group [10,23]. The peak weakening at 1386 cm^−1^ corresponded to the symmetric stretching vibration of COO−, indicating that the carboxyl group was involved in the adsorption process [23]. The spectrum after uranium adsorption shows a characteristic single peak representing uranyl ion near 918 cm^−1^, which also proves again that uranium was successfully adsorbed [24].

As shown in Figure 15b, the C 1s spectrum was deconvoluted into three peaks; the peak at approximately 284.8 eV indicated C=C bonds, the peak at approximately 286.2 eV indicated C−O bonds, and the peak at approximately 288.1 eV indicated C=O bonds [48]. After adsorption, the binding energies of these carbon-containing functional group peaks were reduced to different degrees, among which the C=C and C=O peak intensities were significantly reduced after Th adsorption and the C−O peak intensity was reduced after U adsorption, indicating that they played significant roles in the adsorption processes [32].

**Figure 16:**
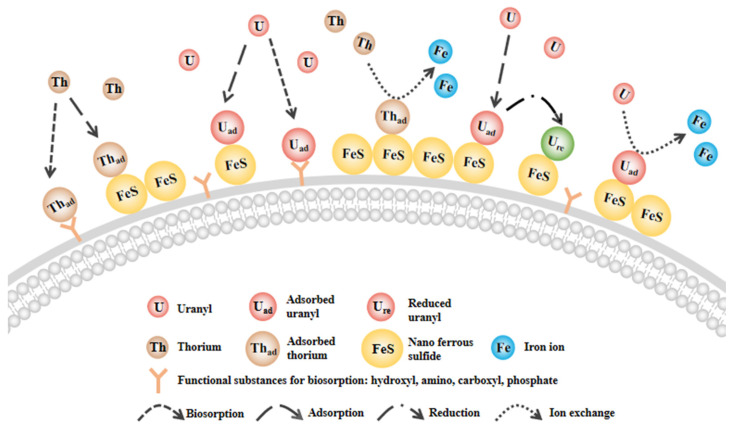
The schematic diagram of FeS-cells removal of radioactive contaminants.

Ion exchange, reduction processes, and biosorption processes are jointly involved in the removal of radioactive elements. The synergy of these three set mechanisms provides new insights into microbial-nanomaterial modification techniques. The mechanism of adsorption of radioactive contaminants by FeS-cells complexes is shown in Figure 16.

## 4. Conclusions

In this study, FeS-cells nano complexes were successfully synthesized by a sonochemical method. The prepared FeS-cells complexes exhibited excellent adsorption of U and Th. The optimal adsorption conditions were as follows: an ultrasonic treatment time of 20 min, a dosage of 0.1 g/L, a pH = 6, 30 °C, and an initial concentration of 50 mg/L. The U and Th adsorption capacities were 481.9 and 407.5 mg/g, which constituted increases of 163% and 142%, respectively, relative to those of the *Stenotrophomonas* sp. cells. The removal mechanisms involved ion exchange, reduction, and microbial complexation, which rendered an excellent removal ability for FeS-cell. The excellent performance greatly broadens the scope for application of the adsorption method and shows that FeS-cells have good prospects for the treatment of radioactive wastewater.

## Figures and Tables

**Table 1:** Parameters of the Langmuir and Freundlich models for FeS-cells adsorption.

Radioactive Element	Langmuir Model			Freundlich Model		
	K_L_	R^2^	Q_e cal_ (mg/g)	K_F_	1/n	R^2^
U^6+^	0.294	0.969	1263.7	351.3	0.375	0.982
Th^4+^	0.128	0.953	610.9	157.9	0.304	0.985

**Table 2:** Kinetic parameters for FeS-cells adsorption.

Radioactive Element	Pseudo-First-Order Kinetic Model				Pseudo-Second-Order Kinetic Model			
	C_0_ (mg/L)	Q_e exp_ (mg/L)	K_1_ (1/h)	R^2^	Q_e cal_ (mg/L)	K_2_ (g/(mg•h))	h (mg/(g•h))	R^2^
U^6+^	50	491.1	0.319	0.875	492.6	0.02145	5204.9	0.999
Th^4+^	50	403.1	0.323	0.505	421.9	0.02174	3869.5	0.999

**Table 3:** Kinetic parameters for intraparticle diffusion during FeS-cells adsorption.

Radioactive Element	C_0_ (mg/L)	Stage 1			Stage 2			Stage 3		
		K_d1_ (mg/(mg·h^1/2^))	C_1_	R^2^	K_d2_ (mg/(mg·h^1/2^))	C_2_	R^2^	K_d3_ (mg/(mg·h^1/2^))	C_2_	R^2^
U^6+^	50	556.4	0	1	109.5	324.6	0.904	5.23	468.2	0.826
Th^4+^	50	419.9	0	1	153	182	0.934	4.55	400.3	0.759

## Data Availability

Not applicable.

## Supplementary Materials

The following supporting information can be downloaded at: https://www.mdpi.com/article/10.3390/toxics11040297/s1, Figure S1: The correlation analysis graph between adsorption capacity and ferrous ion concentration. (a) U (b) Th; Figure S2: XRD patterns of FeS-cells complexes after adsorption. (a) U. (b) Th; Table S1: Uranium adsorption isotherm experimental data. Table S2: Thorium adsorption isotherm experimental data.
