# Supplementary material for: Surface Engineering Design of Nano FeS@Stenotrophomonas sp. by Ultrasonic Chemical Method for Efficient U(VI) and Th(IV) Extraction
Source: Toxics. 2023 Mar 24;11(4):297. doi: 10.3390/toxics11040297 (PMC10144925; doi:10.3390/toxics11040297)
Supplement: Supplementary file 1 [file toxics-11-00297-s001.zip › toxics-2258490-supplementary.pdf]

Supporting Information

Surface Engineering Design of Nano FeS@*Stenotrophomonas* sp. by Ultrasonic Chemical Method for Efficient U(VI) and Th(IV) Extraction

Zhongqiang Hu [1,2], Zhongkui Zhou [1,2,*], Jianping Guo [1,2], Yong Liu [1,2], Shunjing Yang [1,2], Yadan Guo [1,2],

Liping Wang [3], Zhanxue Sun [1,2] and Zhihui Yang [4]

[1] State Key Laboratory of Nuclear Resources and Environment, East China University of Technology, Nanchang 330013, China; hzq15873422787@163.com (Z.H.); gjp18760296196@163.com (J.G.); ly18720133985@163.com (Y.L.); ysj199906@163.com (S.Y.); guoyadan@ecut.edu.cn (Y.G.); zhxsun@ecut.edu.cn (Z.S.)
[2] School of Water Resources and Environmental Engineering, East China University of Technology, Nanchang 330013, China
[3] School of Environmental and Spatial Informatics, China University of Mining and Technology, Xuzhou 221116, China; lpwang@cumt.edu.cn
[4] School of Metallurgy and Environment, Central South University, Changsha 410083, China; yangzh@csu.edu.cn
* Correspondence: zhkzhou80@163.com

1. Adsorption Isotherms

The Langmuir and Freundlich models were chosen to model the adsorption isotherms [15, 22], and they are shown in Equations (1) and (2), respectively.

$$Q_e = Q_{max} \times \frac{K_L C_e}{1 + K_L C_e} \tag{1}$$

$$Q_e = K_F C_e^{1/n} \tag{2}$$

where Q_{max} is the maximum adsorption capacity (mg/g), K_L is the Langmuir constant, which is used to characterize the adsorbent properties, K_F is a constant that characterizes the adsorption capacity and strength and 1/n is the Freundlich constant that characterizes the strength and inhomogeneity of the adsorption system.

2. Adsorption kinetics

The experimental data were fitted using quasi-primary, quasi-secondary, and ion diffusion kinetic models [23]. The kinetic model fitting equations are shown in Equations (3)–(5) [11, 24].

Pseudo-first-order kinetic model:

$$\ln(Q_e - Q_t) = \ln Q_e - K_1 \cdot t \tag{3}$$

Pseudo-second-order kinetic model:

$$\frac{t}{Q_t} = \frac{1}{K_2 \cdot Q_e^2} + \frac{t}{Q_e} \tag{4}$$

$$h = K_2 \cdot Q_e^2 \tag{5}$$

Intra-particle diffusion model:

$$Q_t = K_3 \cdot t^{1/2} + C_i \tag{6}$$

where Q_t is the amount of FeS-cell adsorbed at the moment t (h) (mg/g). K_1 and K_2 are the quasi-primary adsorption rate constants (1/h) and quasi-secondary adsorption rate con-

stants (g/(mg•h)), respectively. h is the initial adsorption rate (mg/(g•h)). k_3 is the intraparticle diffusion adsorption rate constant (mg/(mg•$h^{1/2}$)). Ci is the intercept related to the boundary layer thickness (mg/g).

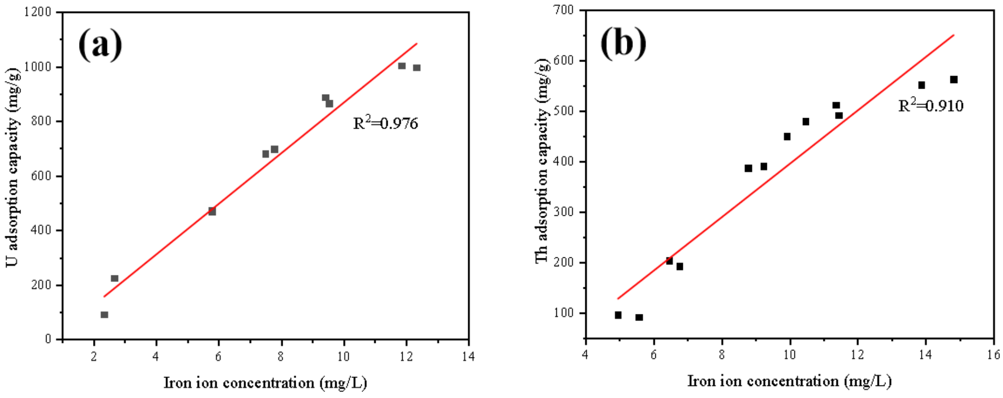

Figure S1. The correlation analysis graph between adsorption capacity and ferrous ion concentration. (**a**) U (**b**) Th.

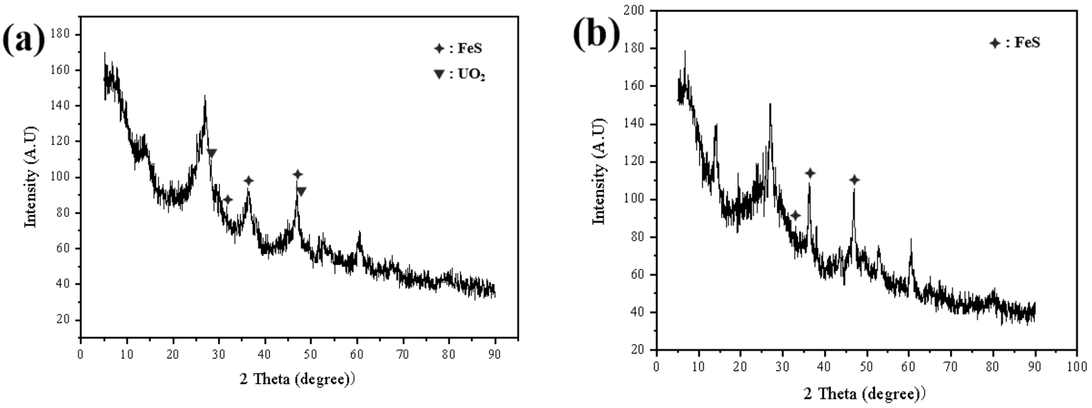

Figure S2. XRD patterns of FeS-cells complexes after adsorption. (**a**) U. (**b**)Th.

Table S1. Uranium adsorption isotherm experimental data.

Uranium Initial Concentration (mg/L)	Uranium Concentration in Solution (mg/L)			C_e (mg/L)	Q_e (mg/g)
10	0.2	0.19	0.21	0.2	98
25	0.55	0.5	0.51	0.52	244.8
50	1.52	1.98	1.9	1.8	482
75	5.41	4.87	4.69	4.99	700.1
100	12.54	10.64	11.44	11.54	884.6
150	29.15	28.39	28.89	28.81	1211.9

Table S2. Thorium adsorption isotherm experimental data.

Thorium Initial Concentration (mg/L)	Thorium True Initial Concentration (mg/L)	Uranium Concentration in Solution (mg/L)			C_e (mg/L)	Q_e (mg/g)
10	9.56	0.22	0.17	0.24	0.21	93.5
25	22.93	3.66	3.18	3.42	3.42	195.1
50	48.11	11.06	11.93	11.7	11.56	365.5
75	70.71	26.74	25.75	26.23	26.24	444.7
100	98.66	47.46	49.53	47.52	48.17	504.9

	150	140.72	81.35	80.75	82.06	81.47	592.5
